# The Chemokine Receptor CXCR4 Mediates Recruitment of CD11c^+^ Conventional Dendritic Cells Into the Inflamed Murine Cornea

**DOI:** 10.1167/iovs.18-25084

**Published:** 2018-11

**Authors:** Maria J. Lopez, Yashar Seyed-Razavi, Arsia Jamali, Deshea L. Harris, Pedram Hamrah

**Affiliations:** 1 Center for Translational Ocular Immunology, Department of Ophthalmology, Tufts Medical Center, Tufts University School of Medicine, Boston, Massachusetts, United States; 2 Schepens Eye Research Institute/Massachusetts Eye and Ear Infirmary, Department of Ophthalmology, Harvard Medical School, Boston, Massachusetts, United States; 3 Program in Immunology, Sackler School of Graduate Biomedical Sciences, Tufts University, Boston, Massachusetts, United States; 4 Cornea Service, New England Eye Center, Department of Ophthalmology, Tufts Medical Center, Tufts University School of Medicine, Boston, Massachusetts, United States; 5 Cornea Service, Massachusetts Eye & Ear Infirmary, Department of Ophthalmology, Harvard Medical School, Boston, Massachusetts, United States

**Keywords:** CXCR4, CXCL12, cornea, antigen presenting cells, conventional dendritic cells

## Abstract

**Purpose:**

The cornea contains distinct populations of antigen-presenting cells (APCs), including conventional dendritic cells (cDCs). Little is known about the molecular mechanisms involved in cDCs homing and recruitment into the naïve and inflamed cornea. The purpose of this study was to investigate the presence of CXCR4 and its ligand CXCL12 in the murine cornea and its role in cDC migration during corneal inflammation.

**Methods:**

The expression of CXCR4 and CXCL12 in naïve and suture-inflamed murine corneas was assessed by whole-mount staining, flow cytometry, and quantitative PCR. The role of CXCR4 in recruitment into inflamed corneas was investigated using adoptive transfer of cDCs blocked with neutralizing antibody against CXCR4.

**Results:**

We show the chemokine receptor CXCR4 to be expressed on 51.7% and 64.8% of total corneal CD11c^+^ cDCs, equating to 98.6 ± 12.5 cells/mm^2^ in the peripheral and 64.7 ± 10.6 cells/mm^2^ in the central naïve cornea, respectively. Along with a 4.5-fold increase in CXCL12 expression during inflammation (*P* < 0.05), infiltrating cDCs also expressed CXCR4 in both the peripheral (222.6 ± 33.3 cells/mm^2^; *P* < 0.001) and central cornea (161.9 ± 23.8 cells/mm^2^; *P* = 0.001), representing a decrease to 31.0% and 37.3% in the cornea, respectively. Further, ex vivo blockade (390.1 ± 40.1 vs. 612.1 ± 78.3; *P* = 0.008) and local blockade (263.5 ± 27.1 vs. 807.5 ± 179.5, *P* < 0.001) with anti-CXCR4 neutralizing antibody resulted in a decrease in cDCs homing into the cornea compared with cells pretreated with isotype controls.

**Conclusions:**

Our results demonstrate that corneal CXCL12 plays a direct role in CXCR4^+^ cDC recruitment into the cornea. The CXCR4/CXCL12 axis is therefore a potential target to modulate corneal inflammatory responses.

The cornea functions as a physical and immunological barrier to the external environment, where it is continuously exposed to foreign particles and pathogens. Concordantly, several active mechanisms dampen corneal immune responses and thus regulate corneal immune privilege, defined by the lack of an immune response to allografts or antigens.^1–4^ The absence of vessels in the cornea also has implications for its immunologic status where, despite the defense mechanisms and immune privilege nature of the ocular surface system, infections and immune-mediated corneal diseases can lead to alterations in the corneal structure, resulting in corneal opacification and subsequent blindness. To date, the standard therapy for corneal immune-mediated diseases is nonspecific immune suppression with steroidal drugs. Few effective anti-inflammatory drugs have emerged over the last decades, and many inflammatory diseases are inadequately responsive to current medications. Hence, there is a clear need to understand molecular mechanisms that drive corneal immune-mediated diseases.

Distinct populations of bone marrow–derived antigen-presenting cells (APCs), such as conventional dendritic cells (cDCs) and macrophages, reside in the naïve cornea.^5–7^ cDCs are professional APCs that capture and process antigens. They function to prime CD4^+^ and CD8^+^ naïve T cells and elicit antigen-specific adaptive immune responses, and have a dual function as key regulators of T cell sensitization and tolerance induction to both self- and foreign antigens.^8–10^ In their immature state, cDCs lack the requisite accessory signals for T cell activation, such as CD40, CD80, and CD86, and remain immature until inflammatory signals in the extracellular milieu induce a rapid change in their function, also known as activation or maturation.^7,11^ During corneal inflammation, the number of cDCs greatly increases, and resident and infiltrating cDCs undergo maturation through increased expression of major histocompatibility complex class II (MHC-II) antigens and upregulation of costimulatory molecules CD40, CD80, and CD86.^11^ Activation and recruitment of cDCs into the cornea has been associated with loss of immune privilege in the anterior segment, such as during exacerbation of infectious keratitis,^12,13^ amplification of corneal transplantation immunity,^14,15^ and dry eye disease.^16,17^ cDC recruitment to the cornea during inflammation is mediated, in part, by the complex interplay between chemokines, their respective receptors, and the multistep adhesion cascade that includes adhesion molecules like selectins and integrins.^18^ Chemokines are a family of chemotactic cytokines, categorized by the presence and particular arrangement of cysteine residues (C, CC, CXC, and CX3C) in their N-terminal region, and are essential in inducing directed chemotaxis and retention of nearby resident or circulating leukocytes.^19^ Therefore, the expression of chemokine receptors on cells and distribution of chemokines in tissues critically influence immune responses. In the normal cornea, constitutive expression of CC chemokine receptors (CCR)-1, -2, and -5 have been described.^20,21^ The partial role of these chemokine receptors in cell recruitment into the inflamed cornea, where receptor expression is upregulated when the corneal homeostasis is disrupted, has been demonstrated.^21,22^ The lack of an absolute effect in disrupting these pathways suggests the involvement of additional receptors, whose chemotactic function in the cornea has not yet been described; one candidate is CXCR4.^23^

CXCR4 is a chemokine receptor first described in 1996 as a fusion and entry cofactor for the human immunodeficiency virus and was initially termed “fusin.”^24,25^ Later, the expression of this receptor was described in hematopoietic progenitor cells and leukemic cells,^26,27^ renal progenitor cells,^28^ and human colonic epithelial cells.^29^ In the eye, expression of CXCR4 has been described on human RPE cells,^30^ choroidal–retinal endothelial cells,^31,32^ limbal epithelial stem cells,^33,34^ and human corneal fibroblasts.^35^ CXCR4 is a known receptor for CXCL12/stromal cell-derived factor (SDF)-1; it has been reported that CXCL12 is a potent chemoattractant involved in immune surveillance.^36^ The CXCR4/CXCL12 axis plays an important role in the migration of lymphocyte subsets, including cDCs and CD4^+^ T cells, in the skin and in blood vessels.^37–39^ Further, CXCR4/CXCL12 engagement has been described as an enhancer of cDC maturation and cell survival.^40^ Little is known about the role of CXCR4 chemokine receptor in the cornea and its chemotactic function; hence, the purpose of this study was to examine the role of the CXCL12/CXCR4 axis in cDC recruitment into the naïve and inflamed corneas. To this end, we performed immunofluorescence staining and flow cytometry analysis of cDCs, as well as adoptive transfer experiments to test the hypothesis that an increase in CXCL12 expression contributes to CXCR4-mediated cDC recruitment during inflamed states of the cornea, whereas blocking this receptor will result in decreased cDC recruitment.

## Methods

### Animals

Eight- to 10-week-old BALB/c mice (Charles River, Wilmington, MA, USA) housed in specific pathogen-free (SPF) facilities were used in all experiments. Animals with corneal abnormalities were excluded from studies. The protocol was approved by the Tufts Medical Center and Schepens Eye Research Institute Animal Care and Use Committees, and all animals were treated according to National Institutes of Health guidelines and the ARVO Statement for the Use of Animals in Ophthalmic and Vision Research.

### Suture-Induced Corneal Inflammation

Animals were anesthetized with a ketamine (100 mg/kg body weight) and xylazine (20 mg/kg) mixture administered intraperitoneally. Once the animals were deeply anesthetized, three interrupted intrastromal sutures (Nylon 11-0 taper point; Surgical Specialties, Wyomissing, PA, USA) were placed to induce corneal inflammation with neovascularization, as previously described.^41,42^ Sutures were placed with two stromal incursions extending 120° of the corneal circumference each. At the end of the procedure antibiotic ointment (Erythromycin Ophthalmic Ointment USP, 0.5%; Bausch & Lomb, Inc., Tampa, FL, USA) was applied to the cornea to reduce the risk of infection. Buprenorphine (0.1 mg/kg) was administered subcutaneously at the time of surgery and every 12 hours for 72 hours to keep the animals pain free during and after recovery.

### Isolation of CD11c^+^ cDCs

Isoflurane-anesthetized naïve mice received a subcutaneous injection of 5 × 10^6^ B16 melanoma cells secreting Flt-3 ligand in the back of the neck (referred to as tumor mice throughout the article) to enhance cDC isolation yield (tumor mice having ∼25 × 10^6^ DCs/ spleen, representing a 40-fold increase in number compared to naïve mice)^43^ and limit the total number of animals required for each experiment. Animals were monitored up to 14 days and euthanized, and splenic cDC single cell suspensions were obtained by positive selection with anti-CD11c microbeads (Cat. 130-097-059; Miltenyi Biotec, Cambridge, MA, USA) following the manufacturer's instructions. Purity of column-isolated cDCs was corroborated with flow cytometry to be greater than 95% CD11c^+^ (Supplementary Fig. S1). The elution of cells was resuspended in MACS buffer (Cat. 130-091-222; Miltenyi Biotec) at a concentration of 25 × 10^6^ cells/0.2 mL.

### Blocking, Labeling, and Adoptive Transfer of cDCs

Isolated CD11c^+^ cDCs were incubated for 30 minutes with a nontoxic concentration (25 μg/mL) of either anti-CXCR4 neutralizing antibody (clone 247506, MAB21651-100; R&D Systems, Minneapolis, MN, USA) or rat IgG2b isotype control (clone 141945, MAB0061; R&D Systems). Additional experiments were conducted without blocking. Next, cDCs were fluorescently labeled with CM-H2DCFDA (CFDA; 1:1000, C6827; Molecular Probes, Eugene, OR, USA) for 30 minutes at 37°C prior to adoptive transfer.

At 7 days after corneal suture placement, adoptive transfer was conducted in three sets of experiments: (1) mice receiving adoptive transfer of 25 × 10^6^ anti-CXCR4–treated or isotype control–treated cDCs via intravenous (i.v.) tail vein injections; (2) mice receiving either anti-CXCR4 neutralizing antibody (50 μg/mL) or isotype control i.v. 30 minutes prior to adoptive transfer of untreated cDCs; and (3) mice that received either anti-CXCR4–neutralizing antibody (25 μg/mL) or isotype control by subconjunctival injections 30 minutes prior to adoptive transfer of untreated cDCs i.v. (*n* = 3 per group per experiment, repeated three times).

### Corneal Confocal Imaging

Twenty-four hours after adoptive transfer of cDCs into sutured corneas, mice were euthanized, corneas carefully excised, fixed in 4% paraformaldehyde (Cat. 15710; Electron Microscopy Sciences, Hatfield, PA, USA) for 20 minutes at room temperature and washed with PBS for 15 minutes. Then, whole corneas were covered with mounting medium including 4′,6-diamidino-2-phenylindole (DAPI; Vector Laboratories, Burlingame, CA, USA) and analyzed with a laser-scanning confocal microscope (Leica TCS SP5; Leica, Heidelberg, Germany).

### Immunofluorescence Staining

Normal and inflamed corneas were harvested, washed in PBS, and fixed in chilled acetone for 15 minutes. To avoid nonspecific staining, corneas were incubated with Fc-block (anti-mouse CD16/32, clone 2.4G2, dilution 1:100; BioXCell, West Lebanon, NH, USA) in 3% BSA diluted in PBS at room temperature for 90 minutes. Corneas were then stained with either anti-CXCR4 primary antibody (clone 247506, Cat. MAB21651-100, dilution 1:50; R&D Systems) and anti-CD11c antibody (conjugated, clone HL3, Cat. 561044, dilution 1:50; BD Bioscience, San Jose, CA, USA), or anti-mouse CXCL12 (Cat. 14-7992-83, 1:100 dilution; eBioscience, San Diego, CA, USA) at 4°C overnight. Next, corneas were incubated for 30 minutes with AlexaFluor 488–conjugated secondary antibody (donkey anti-rat IgG, Cat. A-21208, 1:100 dilution) or AlexaFluor 594–conjugated secondary antibody (donkey anti-rabbit IgG, Cat. 711-585-152, 1:100 dilution; Jackson ImmunoResearch, West Grove, PA, USA). Each staining or incubation was followed by three 5-minute PBS washes. Appropriate controls for CD11c (Armenian hamster IgG, Cat. 400908; Biolegend, San Diego, CA, USA), CXCR4 (rat IgG_2B_, Cat. 400605; Biolegend), and CXCL12 (rabbit IgG, sc-2027; Santa Cruz Biotechnology, Dallas, TX, USA) were performed. Whole corneas were covered with mounting medium including DAPI, and full corneal thickness z-stacks were collected from three regions of the peripheral and para-central cornea each, and one was collected for the central cornea with a laser-scanning confocal microscope and a 40× objective (Nikon A1R Confocal Laser Microscope System, Tokyo, Japan).

### Image Analysis and Quantification

Acquired confocal (*xyz*) stacks of whole-mounted corneas were digitally reconstructed and cropped at the squamous corneal epithelial cell layer to allow for proper quantification of the stroma. CXCR4^+^, CD11c^+^, and CFDA^+^ cells recruited into the corneal stroma after adoptive transfer were counted in a semiquantitative fashion with the spot function of Imaris software (Bitplane, Zurich, Switzerland) based on objective criteria, including morphology, size, and intensity of staining in single- and double- positive analyses. Investigators performing cell density quantifications were blinded. Cell density data are presented as cells/mm^2^. For assessment of cell distribution, the peripheral cornea was defined as the outermost portion of the cornea 1 mm from the corneal limbus, and the combined para-central and central regions of the cornea were considered as the central cornea. CXCL12 distribution throughout the cornea was similarly analyzed with Imaris software.

### Real-Time PCR

RNA from normal and inflamed corneas was isolated using the RNeasy Plus Micro Kit and reverse transcribed using the QuantiTect Reverse Transcription Kit (both from Qiagen, Hilden, Germany). Real-time PCR was performed using iTaq Universal CYBR Green Supermix (Bio-Rad Laboratories, Hercules, CA, USA) and primers for CXCL12α (forward: 5′-GTCAGCCTGAGCTACAGATGC-3′ and reverse: 5′-CACTTTAGCTTCGGGT CAATG-3′) and β-actin (forward: 5′-GTCCTTAATGTCACGCACGATTTC-3′ and reverse: 5′-GTGGGGCGCCCCAGGCACCA-3′)^30^ (Integrated DNA Technologies, Coralville, IA, USA). The results were analyzed using the comparative threshold cycle method and normalized by β-actin as an internal control.

### Flow Cytometry

BALB/c spleens from naïve and tumor mice were mechanically digested and placed through a 70-μm cell strainer to obtain single cell suspensions. To avoid nonspecific staining, single cells were incubated and blocked with 1% Fc-block for 30 minutes at room temperature. Splenocytes were then labeled with fluorophore-conjugated antibodies against CD11c (clone N418, Cat. 117307; BioLegend), CXCR4 (clone 2B11, Cat. 53-9991-80; eBioscience), CD8 (clone 53-6.7, Cat. 100725; BioLegend), CD103 (clone 2-E7, Cat. 121409; BioLegend), and CD11b (clone M1/70, Cat. 101228; BioLegend) or appropriate isotype controls. Following a final wash, stained single cells were fixed with 4% paraformaldehyde (Cat. 15710; Electron Microscopy Sciences) and then analyzed using a BD LSR II Flow Cytometer (BD Biosciences).

Naïve and suture-inflamed BALB/c corneas were harvested and immersed in PBS containing 20 mM EDTA (Sigma-Aldrich) at 37°C for 30 minutes, and the corneal epithelium was removed with forceps to allow investigation of the corneal epithelium and stroma separately. Following two washes with PBS, the corneal stroma was cut into pieces and digested with 2 mg/mL collagenase D (Roche, Indianapolis, IN, USA) and 2 mg/mL DNAse I (Sigma-Aldrich, St. Louis, MO, USA) in DMEM for 60 minutes at 37°C. After digestion, corneal single cell suspensions were passed through a 70-μm cell strainer, and corneal epithelia and stroma were pooled into one and two separate samples per state, respectively, and blocked with Fc-block (as above). Samples were stained with fluorophore-conjugated antibodies against cell surface markers CXCR4 (2B11, Cat. 53-9991-80; eBioscience), CD45 (30-F11; BioLegend), CD11c (HL3; BD Biosciences), F4/80 (BM8; BioLegend), or appropriate conjugated isotype controls. Single cell suspensions were then washed, fixed, and permeabilized (Cat. 555028; BD Bioscience) and labeled with CXCL12 (Cat. 14-7992-83; eBioscience). Single cell suspensions were then stained with an anti-rabbit IgG secondary antibody (Jackson ImmunoResearch), and following a final wash underwent flow cytometric analysis using BD LSR II Cytometer (BD Biosciences). Acquired data were analyzed by FlowJo v10 (FlowJo, LLC, Ashland, OR, USA). Data are presented as relative expressions compared with isotype controls.

### Statistical Analysis

Results are presented as mean ± SEM, and statistical significance was determined for each by either two-tailed Student *t*-test or 1-way ANOVA with a Tukey post hoc test (Prism Graphpad Software, La Jolla, CA, USA) to correct for multiple comparisons made during the ANOVA. Differences between groups were considered significant at *P* < 0.05.

## Results

### CXCR4 in the Naïve and Inflamed Cornea

**Figure 1:**
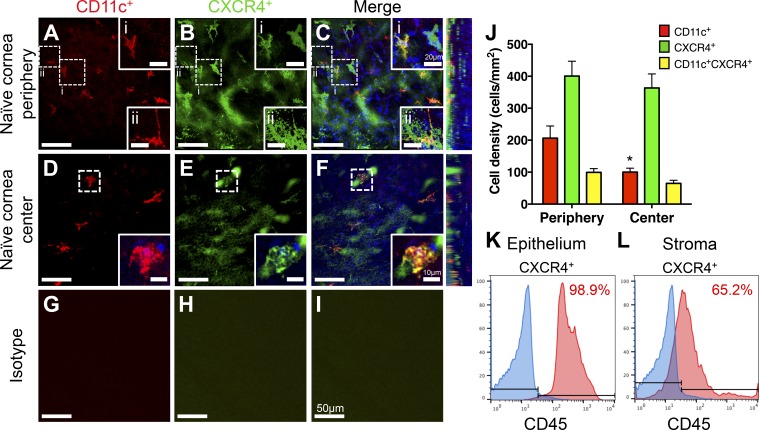
CXCR4 chemokine receptor is constitutively expressed in the naïve murine cornea. Representative confocal micrographs of naïve BALB/c corneas in peripheral (A–C) and central (D–F) stroma show expression of CD11c^+^ (red, A, D) and CXCR4^+^ (green, B, E) and colocalization (yellow, C, F) throughout the corneal stroma. Orthogonal views highlighting positive staining added to each corneal area. White dotted boxes show examples of CD11c^+^ DCs (magnified in A–C, stroma (i) and epithelium (ii); and D–F insets), as well as a CD11c^−^ cells (magnified in A–C, inset i) coexpressing CXCR4 in the peripheral and central corneas. Isotype controls for CD11c and CXCR4 do not show positive staining (G–I). Cell density quantification for CD11c^+^ and CXCR4^+^ cells (cells/mm^2^) highlights populations of steady-state cDCs expressing CXCR4: significance for cell densities between central and peripheral cornea is denoted by an asterisk (J). Representative histograms of flow cytometry analysis of relative CXCR4 expression of CD45^+^ leukocytes in the inflamed corneal epithelium (K) and stroma (L). Data are shown as mean ± SEM. t-test, *P < 0.05. Scale bars denote 50 μm (A–I), 20 μm (A–C, insets i and ii), and 10 μm (D–F insets). n = 4.

The presence and distribution of a diverse population of APCs, including cDCs, within the cornea have previously been described in detail.^5–7^ cDCs, which have been shown to constitutively express the chemokine receptor CXCR4,^23^ are recruited to the cornea during inflamed states. Thus, we sought to investigate the role of CXCR4 in corneal cDC recruitment. To assess whether steady-state corneal cDCs express CXCR4, we performed whole-mount immunofluorescence imaging of naïve corneas with anti-CD11c and anti-CXCR4 monoclonal antibodies. We found CXCR4 to be constitutively expressed throughout the corneal epithelium, more notably in the peripheral corneas (Figs. 1A–1C), as well as within the corneal stroma in both peripheral and central corneas (Figs. 1A–1F). Examples of CD11c/ CXCR4 double-labeled cDCs within the corneal stroma (Figs. 1A–1C, insert i, and 1D–1F) and epithelium (Figs. 1A–1C, insert ii) could be noted in both en face and orthogonal views of whole-mounts. No staining was observed with isotype controls (Figs. 1G–1I). Quantification of whole-mounts revealed cDC density to be significantly different between the central and peripheral corneas (100.2 ± 12.4 compared with 206.2 ± 38.2 cell/mm^2^; *P* < 0.05). No significant difference in CXCR4^+^ (363.5 ± 43.9 compared with 378.4 ± 53.5; *P* = 0.84) and CD11c^+^CXCR4^+^ (64.8 ± 10.6 compared with 98.6 ± 12.5; = 0.06) was noted between the central and peripheral corneas, respectively (Fig. 1J). The expression of CXCR4 by CD11c^+^ cDCs represented 51.7% (range, 28.6% to 75.0%) and 64.8% (range, 33.3% to 85.7%) of total corneal CD11c^+^ cDCs, respectively. Flow cytometry analysis of the corneal epithelium and stroma revealed that, of CXCR4^+^ cells in the epithelium, 98.9% of CXCR4 signal originated from CD45^+^ leukocytes (Fig. 1K), whereas CXCR4 expression was noted in 65.2% of corneal leukocytes (Fig. 1L).

**Figure 2:**
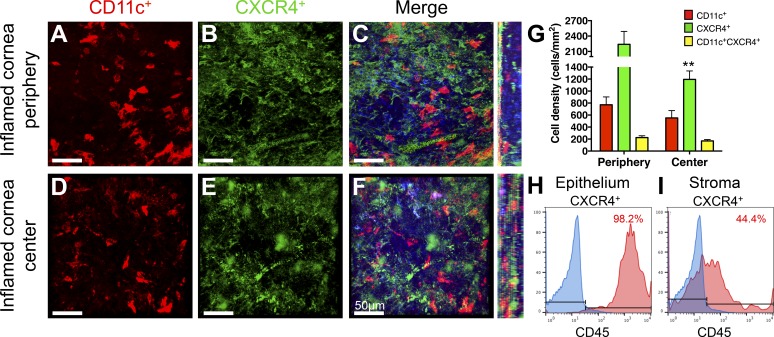
CXCR4 chemokine receptor expression increase in the inflamed murine cornea. Representative confocal micrographs of 7-day suture inflamed BALB/c corneas show increased CD11c^+^ cells (red, A, D), CXCR4^+^ cells (green, B, E), and colocalization (yellow, C, F) throughout the corneal stroma of the peripheral (A–C) and central corneas (D–F). Orthogonal views highlight distribution throughout each corneal area. Cell density quantification of CD11c^+^ and CXCR4^+^ cells in peripheral and central corneal stroma (cells/mm^2^) highlights populations of CXCR4^+^ cDCs to be present following inflammation: significance for cell densities between central and peripheral cornea is denoted by asterisks (G). Representative histograms of flow cytometry analysis of relative CXCR4 expression within CD45^+^ leukocytes in the inflamed corneal epithelium (H) and stroma (I). Data are shown as mean ± SEM. t-test, **P < 0.01. Scale bars denote 50 μm. n = 4.

During corneal inflammation, there was an increase in corneal APCs.^7,11^ As such, we anticipated that expression of CXCR4 will also increase after placement of intrastromal sutures to induce corneal inflammation. Seven days after suture placement, we found an increase in CXCR4^+^ cells throughout the cornea (Figs. 2A–2F). Quantification of inflamed whole-mounts revealed a significant increase in cDC density (central: 552.7 ± 122.1, *P* < 0.001; peripheral: 771.6 ± 130.4, *P* = 0.001), CXCR4^+^ density (central: 1194 ± 141.1, *P* < 0.001; peripheral: 2239 ± 252.6, *P* < 0.001), and CD11c^+^CXCR4^+^ density (central: 161.9 ± 23.8, *P* = 0.001; peripheral: 222.6 ± 33.3, *P* < 0.001; Fig. 2G), compared with steady-state. Comparing between central and peripheral areas of the inflamed cornea, significance was only noted in CXCR4^+^ cell density (1194 ± 141.1 vs. 2239 ± 252.6; *P* = 0.002; Fig. 2G). CD11c^+^CXCR4^+^ density values indicated that 31.0% (range, 13.8% to 64.7%) and 37.3% (range, 21.2% to 88.9%) of corneal cDCs coexpressed CXCR4 in the peripheral and central corneas following inflammation, respectively. Flow cytometry analysis of the inflamed corneal epithelium and stroma revealed similar results to the steady-state, with 98.2% of epithelial (Fig. 2H) and 44.4% of stromal cells expressing CXCR4 found to originate from CD45^+^ leukocytes (Fig. 2I).

### CXCL12 Expression in the Naïve and Inflamed Cornea

**Figure 3:**
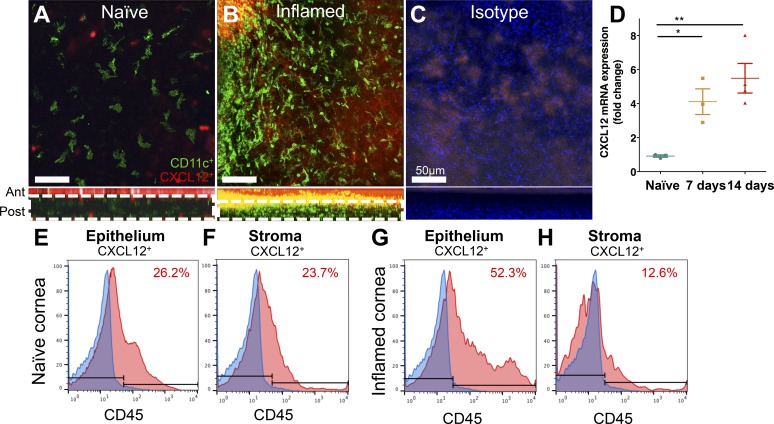
CXCL12 expression in the naïve and inflamed cornea. Representative confocal micrograph of naïve (A) and inflamed (B) BALB/c corneas, with the respective isotype control (C), show expression of CD11c^+^ cDCs (green) and CXCL12 (red) within the corneal stroma. The orthogonal views reveal CXCL12 intensity from the naïve and inflamed corneal epithelium. Further, the white dotted box denoted within corresponding orthogonal views below each en face image highlights the area presented in the top panel images. CXCL12 mRNA expression is upregulated in the cornea 7 and 14 days after suture placement compared with naïve corneas (D). Representative histograms of flow cytometric analysis of CXCL12 expression within CD45^+^ leukocytes in the naïve (E) and inflamed (F) corneal epithelium and stromal layers. Scale bars denote 50 μm. Data are shown as mean ± SEM. *P < 0.05, **P < 0.01. n = 3/setting.

CXCR4 is a known receptor for CXCL12. We therefore next sought to assess the expression of CXCL12 in the cornea. Whole-mount immunofluorescence staining revealed CXCL12 signal throughout the epithelium of both naïve and inflamed corneas (Figs. 3A, 3B, orthogonal view), whereas CXCL12 staining was only observed within the stroma of inflamed corneas (Figs. 3A, 3B, the epithelial signal optically removed in en face images to highlight stromal expression, area denoted by white dotted boxes in orthogonal views). The expression of CXCL12 in the stroma during inflammation could be both due to de novo expression of CXCL12 in the stroma or due to migration of CXCL12-positive cells into the stroma. No staining was observed with isotype controls (Fig. 3C). We next sought to assess *CXCL12* gene expression alteration in the inflamed cornea. *CXCL12* expression was significantly upregulated 7 days (4.5-fold increase; *P* < 0.05) and 14 days (5.9-fold increase; *P* < 0.01) following induction of inflammation by suture placement compared with naïve corneas (Fig. 3D). CXCL12 levels were also investigated by flow cytometry. CXCL12 mean fluorescence intensity (MFI) shifted slightly from 1079 during steady-state to 1203 in the epithelium following inflammation; cellular expression of CXCL12 in the stroma was minimal. Further analysis revealed CD45^+^ corneal leukocytes were responsible for 22.6% and 15.9% (range, 14.9% to 16.8%) CXCL12 expression in the epithelium and corneal stroma under steady-state conditions, respectively (Fig. 3E). Following inflammation, leukocyte expression of CXCL12 increased to 37.4% in the corneal epithelium; however, leukocyte CXCL12 expression remained low at an average of 7.54% (range, 6.79% to 8.29%) in inflamed stromal CD45^+^ leukocytes (Fig. 3F).

Taken together, these results show that CXCL12 is expressed in the naïve cornea, that steady-state corneal cDCs express the chemokine receptor CXCR4, and that during corneal inflammation there is an upregulated expression of both ligand and receptor.

### CXCR4 Expression in Splenic cDCs

**Figure 4:**
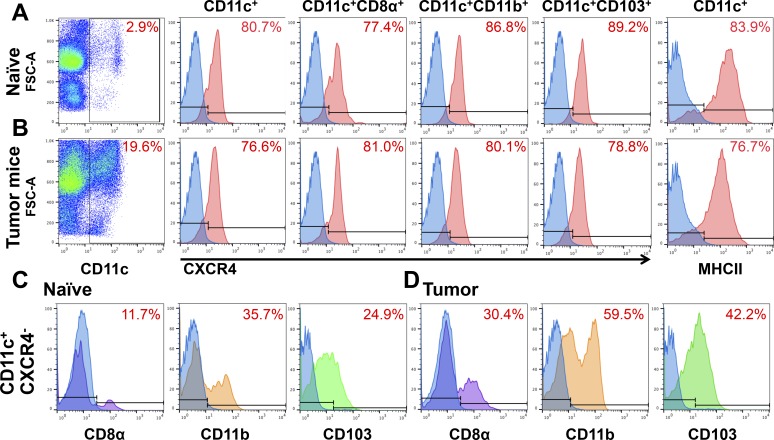
cDCs express CXCR4. Representative flow cytometry plots of CD11c^+^ cDCs from three spleens including cDC percentage of total splenocytes, expression of chemokine receptor CXCR4 in total cDCs and cDC subpopulations (CD8α^+^, CD11b^+^, and CD103^+^), and the cDC maturation marker MHC-II, respectively. CXCR4 was present in all CD8α^+^, CD103^+^, and CD11b^+^ cDC subsets, with similar expression in spleens from naïve BALB/c wild-type (A) and tumor mice (B). Analysis of CXCR4^–^ splenic CD11c^+^ cDCs from wild-type (C) and tumor mice (D).

The next step in investigating the CXCR4/CXCL12 axis in the cornea was to highlight the functional role of this axis by performing adoptive transfer experiments. To determine the feasibility of using splenic cDCs for adoptive transfer experiments and to explore potential differences between cDCs of naïve and FLT-3 ligand-secreting tumor mice, we assessed CXCR4 expression and the maturation status of splenic cDCs. BALB/c splenocytes from naïve and tumor mice were analyzed by flow cytometry and the expression of CXCR4 receptor in total cDCs and cDC subpopulations (CD8α^+^, CD11b^+^, and CD103^+^),^44,45^ and prevalence of mature (MHC-II^+^) cDCs was assessed. Analysis of splenocytes from naïve mice revealed 80.7% of cDCs express CXCR4. Further, CXCR4 expression was noted in 77.4% of CD8α^+^, 86.8% of CD11b^+^, and 89.2% of CD103^+^ cDCs. Analysis of the maturation state of naïve splenic cDCs revealed 83.9% expressing the maturation marker MHC-II (Fig. 4A). We next sought to investigate whether CXCR4 expression is altered in splenocytes from FLT-3 ligand-induced tumor mice. Aside from enhanced cDC isolation yield, CXCR4 expression within total cDCs remained similar between tumor and naïve mice, with 76.6% of splenic cDCs from tumor mice expressing CXCR4 (Fig. 4B). Analysis of cDC subsets revealed CXCR4 is expressed in 81.0% of CDα^+^, 80.1% of CD11b^+^, and 78.8% of CD103^+^ cDCs. The maturation state of splenic cDCs also did not change in tumor mice, with 76.7% of cDCs expressing the maturation marker MHC-II (Fig. 4B). Taken together, these results show that the CXCR4 chemokine receptor is expressed by splenic cDCs, present in all three cDC subsets, and that the maturation state and CXCR4 expression within splenocytes from FLT-3 ligand-secreting tumor mice are comparable to naïve mice.

We next sought to assess the subsets of splenic cDCs that did not express CXCR4. Of total splenic CD11c^+^ cDCs that were CXCR4^−^, 11.7% were CD8α^+^, 35.7% were CD11b^+^, and 24.9% were CD103^+^ (Fig. 4C). FLT-3 ligand-induced tumor mice resulted in an increase in all splenic cDC resident and migratory subsets negative for CXCR4, with 30.4% of CXCR4^−^ cDCs being CD8α^+^, 59.5% were CD11b^+^, and 42.2% were CD103^+^ (Fig. 4D).

### Anti-CXCR4 Treatment Alters cDC Recruitment Into Inflamed Corneas

**Figure 5:**
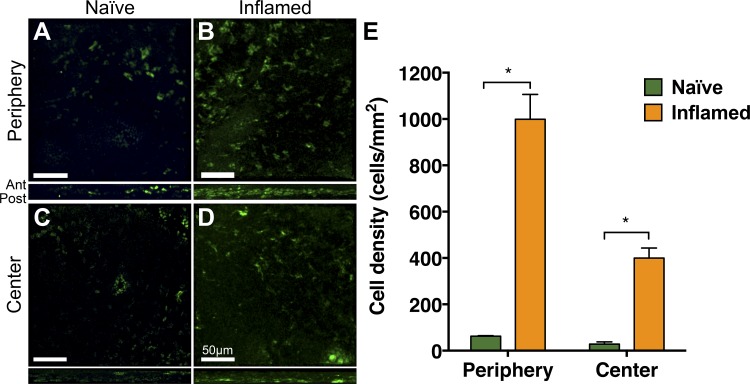
Inflammation leads to increased recruitment of adoptively transferred cDCs into the cornea. Representative confocal micrographs of the peripheral (A, B) and central (C, D) corneas and respective orthogonal views, highlighting distribution of recruited cDCs throughout the cornea of naïve (A, C) and suture inflamed (B, D) BALB/c corneas 24 hours following intravenous adoptive transfer of fluorescently labeled cDCs (green). (E) Cell density quantification of recruited cDCs into the corneal stroma (density cells/mm^2^). Scale bars denote 50 μm. Data are shown as mean ± SEM. *P < 0.05. n = 4/group.

We first sought to assess whether adoptively transferred cDCs are recruited to naïve corneas. Accordingly, 25 × 10^6^ isolated cDCs were fluorescently labeled and injected i.v. into naïve mice. Twenty-four hours after injection, fluorescent cDCs were found in the cornea (42.9 ± 8.8 cells/mm^2^). Stratifying by area, we found cDCs were distributed predominantly in the periphery (Fig. 5A) and in a lesser amount in the center (Fig. 5C). We next assessed recruitment of adoptively transferred cells into the 7-day suture inflamed cornea. A significantly increased recruitment of cDCs was noted in inflamed corneas (670.5 ± 64.1 cells/mm^2^; *P* = 0.004 compared with naïve corneas). Stratified by area, we noted the significant increase in cDCs to be in both the peripheral (998.8 ± 107.5 vs. 62.3 ± 2.3 cells/mm^2^; *P* = 0.01; Fig. 5B) and central regions (399.5 ± 43.3 vs. 28.5 ± 9.9 cells/mm^2^; *P* = 0.01; Fig. 5D) of inflamed corneas compared with naïve corneas (Fig. 5E).

**Figure 6:**
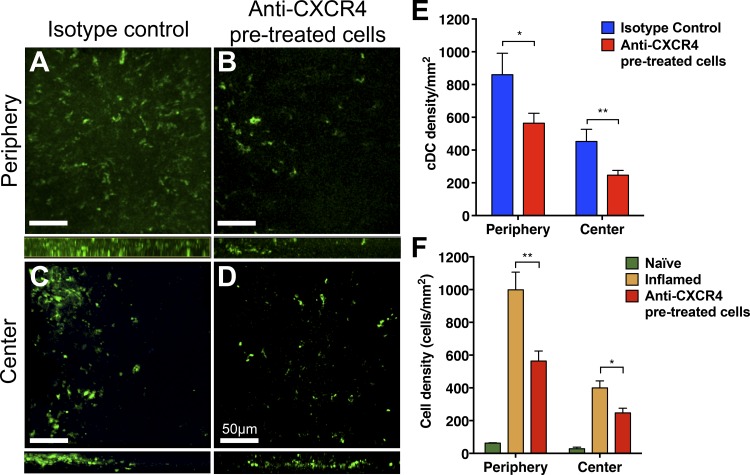
CXCR4 blockade decreases the recruitment of adoptively transferred cDCs. Pretreatment of adoptively transferred cDCs with anti-CXCR4–neutralizing monoclonal antibody reduces infiltration of adoptively transferred cDCs into the peripheral (A, B) and central (C, D) cornea 24 hours after injection compared with isotype control treatment, n = 7/group. Density quantification of cDC infiltration treated ex vivo with anti-CXCR4–blocking antibody reveal a significant decrease in the number of adoptively transferred cells compared with the appropriate isotype control (E) and total infiltration (F) into suture inflamed corneas. Scale bars denotes 50 μm. Data are shown as mean ± SEM. *P < 0.05, **P < 0.01. n = 4–7/group.

Having confirmed recruitment of adoptively transfered cDCs to the cornea, we next investigated the functional role of the CXCR4/CXCL12 axis in cDC recruitment to the cornea. Accordingly, cDCs were first pretreated for 30 minutes with either anti-CXCR4 monoclonal antibody or with isotype control prior to adoptive transfer into mice with inflamed corneas (7 days after corneal sutures placement). Twenty-four hours following adoptive transfer, mice were euthanized, and corneas were analyzed by confocal microscopy to assess cDC recruitment. A significant decrease in recruitment of labeled cDCs into the cornea was noted in the CXCR4-treated group (390.1 ± 40.1 vs. 612.1 ± 78.3, *P* = 0.008) compared with the isotype control group. Stratified by area, a significant difference was noted in both peripheral and central corneas (periphery: 563.8 ± 60.8 vs. 860.1 ± 130.9 cells/mm^2^, *P* = 0.03; center: 247.0 ± 28.9 vs. 452.8 ± 74.0 cells/mm^2^, *P* = 0.007) compared with isotype controls (Figs. 6A–6E).

In line with a relevant treatment option that is clinically translatable to human therapy, we sought to investigate the efficacy of both systemic and local anti-CXCR4 treatment. Accordingly, anti-CXCR4 or isotype control was administered either i.v. or subconjunctivally 30 minutes prior to adoptive transfer, and recruitment of labeled cDCs into the inflamed cornea was investigated 24 hours later. We found that systemic treatment only reduced recruitment to the periphery area, with near significant differences with a single anti-CXCR4 antibody injection (586.1 ± 97.8 vs. 982.5 ± 172.5 cells/mm^2^; *P* = 0.06), whereas no difference was noted within the central cornea (328.9 ± 58.6 vs. 324.5 ± 55.8 cells/mm^2^; *P* = 0.96; Supplementary Fig. S2A). Local subconjunctival injection of neutralizing antibody, however, resulted in a significant decrease in cell recruitment to the inflamed cornea (263.5 ± 27.1 vs. 807.5 ± 179.5; *P* < 0.001). Stratified by area, significant decrease in recruitment was noted both in the peripheral (343.9 ± 35.4 vs. 1264 ± 298.0 cells/mm^2^; *P* = 0.001) and central (206.7 ± 33.2 vs. 401.4 ± 94.3 cells/mm^2^; *P* = 0.03) inflamed cornea compared with animals treated with isotype control (Supplementary Fig. S2B).

Our data suggest a distinct role for CXCR4 in cDC recruitment to the inflamed cornea and highlight local anti-CXCR4 treatment as a potentially clinically viable therapeutic avenue for the treatment of corneal inflammatory disorders.

## Discussion

Inflammatory responses to injury, noxious stimuli, or the invasion of opportunistic bacterial and viral pathogens that penetrate the physical barrier of the ocular surface can result in collateral structural damage to the cornea and lead to corneal opacity and ultimately vision impairment. The mammalian cornea contains heterogeneous populations of bone marrow–derived resident immune cells, including APCs, which significantly increase in density following ocular inflammation.^5,7,11,46,47^ The underlying complex mechanisms behind this increase, however, remain unclear. The majority of steady-state APCs that populate the naïve cornea, especially in the central cornea, phenotypically align with an “immature” subtype in that they express none to minimal levels of MHC-II and co-stimulatory molecules.^48,49^ As APCs function in both the innate and adaptive immune response, described as the initiators and modulators of the immune response,^8,9^ their alteration following corneal injury including maturation, activation, and migration is imperative to limit the collateral damage that often leads to an overactive immune response.

In an inflammatory setting, resident cells secrete chemokines, which in turn direct leukocyte migration and placement in tissues by binding to the associated chemokine receptor(s) on the cell surface or binding to the extracellular matrix where they can remain and attract and retain leukocytes. Examples of chemokine receptors playing a pivotal role in APC recruitment and maintenance within an inflammatory site include CCR1, 2, and 5.^50–53^ The expression profile of CCR1, 2, and 5 in the normal cornea and their role in cDC recruitment into the inflamed cornea have been described.^20,21,22^ However, analysis of chemokines and CC chemokine receptors in the cornea after cautery revealed a significant decrease in recruitment of MHC-II^+^ epithelial cDCs to the central cornea in CCR5 knockout (KO) mice but not in CCR1 KO, CCR2/CCL3 (macrophage inflammatory protein 1α [MIP]-1α) KO, or CCL3 KO mice.^22^ Another study revealed CX3CR1 to be important specifically for the normal recruitment of MHC-II^+^ putative Langerhans cells to the corneal epithelium.^54^ Interestingly, investigating the effect of CCR1 and CCR5 in corneal allograft survival, we found no improved survival for corneas transplanted into CCR2/CCL3 double KO or CCR5 KO mice, whereas grafts into CCR1 KO hosts had a more favorable survival rate (60%) compared with control wild-type mice (20%).^21^ Further, the study showed a significant decrease (>50%) in the number of infiltrating leukocytes in corneas grafted into CCR1 KO mice.

The CXCR4/CXCL12 axis has been described as an important pathway involved in hematopoietic stem and progenitor cells mobilization,^55–57^ migration of leukocyte subsets like cDCs,^36,39^ and cDC survival and maturation to initiate immune responses.^40^ Moreover, the presence of CXCL12 has been described on DCs in the human skin,^58^ leading to the possibility that CXCR4/CXCL12 axis could be a player in corneal cDC recruitment. In mice, CXCR4 has been described as a receptor on cutaneous DCs, with CXCL12 being produced by lymph vessels of the skin and upregulated following antigen exposure, leading to cDC migration toward lymphatic vessels.^39^ In our analyses of CXCL12 expression, we did not note any positive signal originating from vascular structures. In the cornea, CXCR4 has previously been described in murine corneal epithelial cells,^59^ cultured human corneal fibroblasts,^35^ and its functionality activity assessed by calcium mobilization after stimulation with CXCL12.^35^ Whereas our whole-mount immunofluorescence data indicated potential CXCR4 expression originating from the corneal epithelial cells, our flow cytometric analysis indicates the majority of CXCR4 expression in the corneal epithelium to be localized to epithelial leukocytes. Further, we revealed CXCR4 expression in nonleukocytes within the corneal stroma. We also noted CXCL12 expression to increase during corneal inflammation, with our whole-mount corneal immunofluorescence staining data revealing an increase in signal from the corneal epithelium and a clear increase in the extracellular CXCL12 signal. This mirrors other reports indicating that, during systemic inflammation, there is an increase in splenic CXCR4 and CXCL12 mRNA, cell surface and protein expression,^60^ and, specifically in inflamed states of the cornea, there was an increase in gene expression of other inflammatory chemokines, such as regulated on activation, normal T-cell expressed and secreted (RANTES), CCL3, CCL4 (MIP-1β), and CXCL2 (MIP-2), leading to an increase in cell influx.^22^

Whereas the CXCR4/CXCL12 pathway in DC homing to the cornea has not been directly investigated, Cook et al.^61^ found elevated corneal CXCR4 gene expression in parallel to CCR1, 2, and 5, 3 days after infection when examining the expression profile of chemokines (including CCL3/4, RANTES, and monocyte chemoattractant protein-1 [MCP]-1), as well as chemokine receptors (including CCR1-7 and CXCR4) in a murine model of herpes simplex virus (HSV)-1 infection. Moreover, the similarity of the “induction ratio” (relative expression in HSV-infected/relative expression in sham-infected cornea) to that of the other chemokine receptors reported in their paper is interesting to note, as it indicates an increase in CXCR4 following scarification in the sham group.^61^ Our results revealed that approximately 60% of steady-state cDCs in the naïve cornea were CXCR4 positive. This percentage decreased to an average of 35% of total cDCs following suture placement. Moreover, our data showed that both resident (CD11c^+^CD8^+^ and CD11c^+^CD11b^+^) and migratory (CD11c^+^CD103^+^) splenic cDC subsets expressed the CXCR4 chemokine receptor, making this receptor a potential target to modulate cDC migration and recruitment into nonlymphoid tissues. Although the effect of CXCR4 blockade on infiltration of CXCR4-negative DCs to the inflamed cornea was not investigated, any effect would likely be indirect due to the decrease in CXCR4-positive infiltration or general decrease in inflammatory cytokines and chemokines. Further, CXCR4 expression within immunogenic (CD11b^+^CD103^−^) and tolerogenic (CD11b^−^CD103^+^) cDCs infiltrating to the cornea, based on the presence of CD103, warrants further investigation. A limitation of our method of investigating the CXCR4 pathway is that the competitive homing assay does not factor in host DC recruitment and in turn may lead to the underestimation of CXCR4-mediated total cDC recruitment. Further, the impact of disruption of ocular bacteria due to mice being housed within an SPF environment, shown to affect the immune signature of the ocular surface,^62^ on the expression pattern of CXCR4/CXCL12 and DC recruitment was not investigated in this study but may theoretically impact baseline DC density.

CXCL12 expression has been noted in type I muscle fibers,^63^ human skin endothelial cells,^58^ medullary region of lymph nodes,^64^ cells tightly associated with LYVE-1^+^ lymphatic vessels,^39^ and primary human skin fibroblasts (not keratinocytes).^65^ Of note, several studies report CXCL12 expression to be very low in steady-state, increasing following inflammation/damage.^39,61,63,65^ Our data are in accordance with these reports, where the levels of CXCL12 expression in the corneal epithelium and stroma increased following suture placement.

An important aspect of our results is the potential use of CXCR4 as a target to modulate corneal immune responses in patients. We demonstrate that local anti-CXCR4–neutralizing antibody treatment resulted in a decrease in cDC homing to the inflamed cornea, suggesting a potential role of topical drugs targeting this chemokine receptor in human therapies in which patient's cDCs cannot be obtained to be treated. Although our single systemic treatment only led to a near significant difference in cDC homing, multiple or continuous dosage, as well as increased concentration of CXCR4 blockage, may be required to effectively alter cDC infiltration into the inflamed cornea. This is the first report on the implication of CXCR4 blockade in cDC homing to the cornea in a murine model of keratitis. Further longitudinal investigations into the clinical implications of CXCR4 blockade can now be undertaken in relevant disease models, but are beyond the scope of this more mechanistic paper.

Taken together, our results show that the CXCR4/CXCL12 axis plays a significant and functional role in cDC recruitment during corneal inflammation. This is consistent with published data, highlighting the chemotactic properties of CXCL12 in vitro and decreased cutaneous cDC migration into draining lymph nodes in the skin following CXCR4 blockade in vivo.^39^ However, the CXCR4/CXCL12 axis is not the only pathway for cDC infiltration into the cornea. Accordingly, CXCR4 antagonism did not result in complete inhibition of cDC recruitment. Nevertheless, our findings are similar to the study by Kabashima et al.,^39^ in which treatment with a CXCR4 antagonist resulted in partial (approximately 60%) yet significant impairment of cutaneous cDC migration. Worth noting are the subsets of cells in the corneal stroma expressing CXCR4 that were CD11c^−^, that may include corneal keratocytes,^65^ and CD11c^+^ cells that do not express CXCR4 either due different populations with various phenotypic and activation states or infiltrating cDCs downregulating their expression of CXCR4 following entry into the cornea: a chemokine receptor switch.^66^ The effect of combined blockade of chemokine pathways, including CCR2 and CCR7, implicated in DC migration from the skin^67–69^ and cornea^70–72^ to the lymph nodes, will likely be required to significantly impede corneal inflammation, the implications of which will be paramount for modulating immune responses during corneal immune-mediated diseases. In conclusion, our data suggest the role of CXCR4/CXCL12 axis in recruitment of cDCs to the inflamed cornea, making these a potential target to modulate inflammatory responses in murine corneas.

## Supplementary Material

Supplement 1

Supplement 2
