# Supplementary material for: The Chemokine Receptor CXCR4 Mediates Recruitment of CD11c+ Conventional Dendritic Cells Into the Inflamed Murine Cornea
Source: Invest Ophthalmol Vis Sci. 2018 Nov;59(13):5671–81. doi: 10.1167/iovs.18-25084 (PMC6266730; doi:10.1167/iovs.18-25084)
Supplement: Supplement 1 [file iovs-59-13-22_s01.pdf]

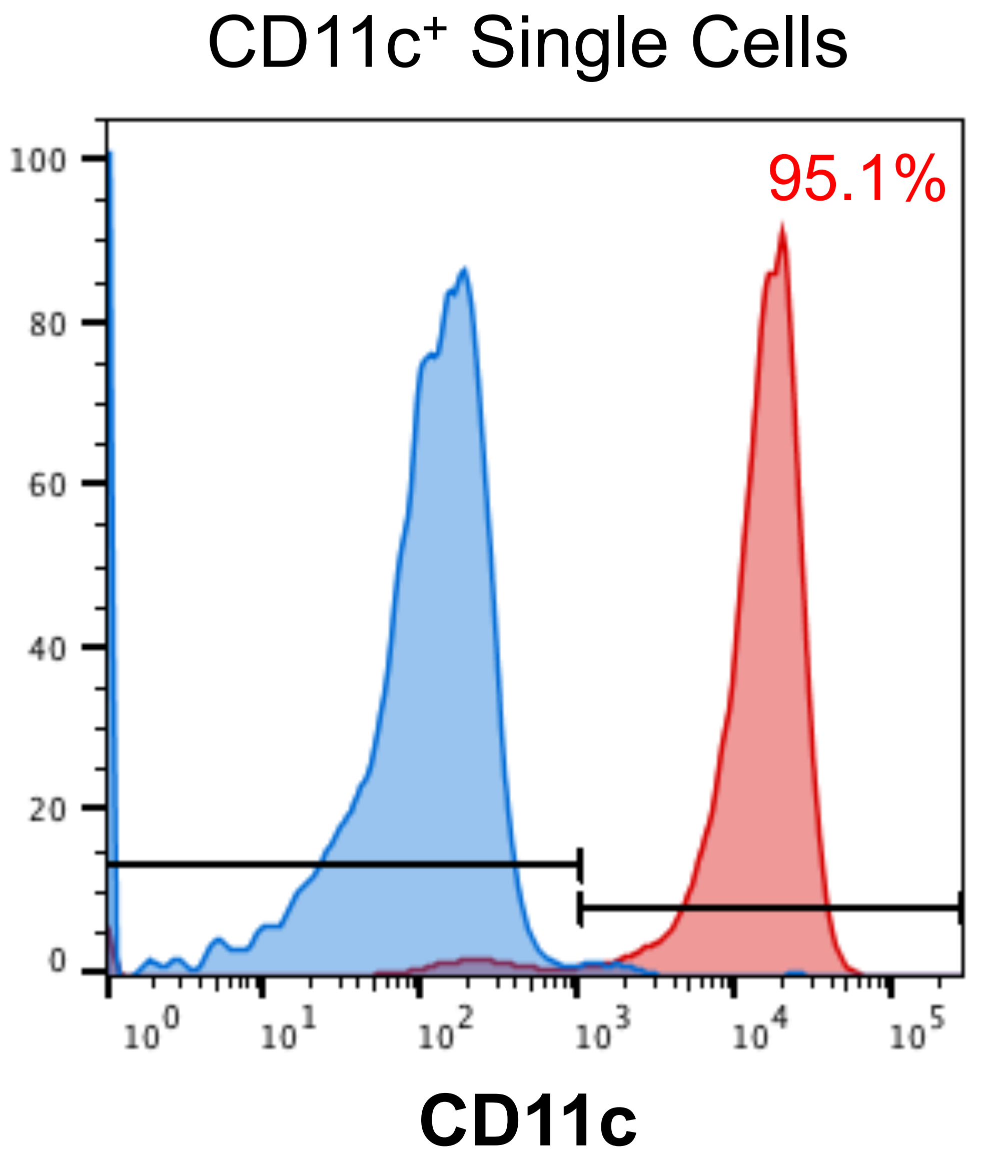
CD11c+ Single Cells
95.1%
100
80
60
40
20
0
10^0
10^1
10^2
10^3
10^4
10^5
CD11c

Supplemental Figure 1. Purity of CD11c$^+$ cells isolation. Utilizing anti-CD11c magnetic microbeads, the purity of column sorted CD11c$^+$ cDCs was found to be greater than 95% by flow cytometric analysis.
