# Supplementary material for: The Chemokine Receptor CXCR4 Mediates Recruitment of CD11c+ Conventional Dendritic Cells Into the Inflamed Murine Cornea
Source: Invest Ophthalmol Vis Sci. 2018 Nov;59(13):5671–81. doi: 10.1167/iovs.18-25084 (PMC6266730; doi:10.1167/iovs.18-25084)
Supplement: Supplement 2 [file iovs-59-13-22_s02.pdf]

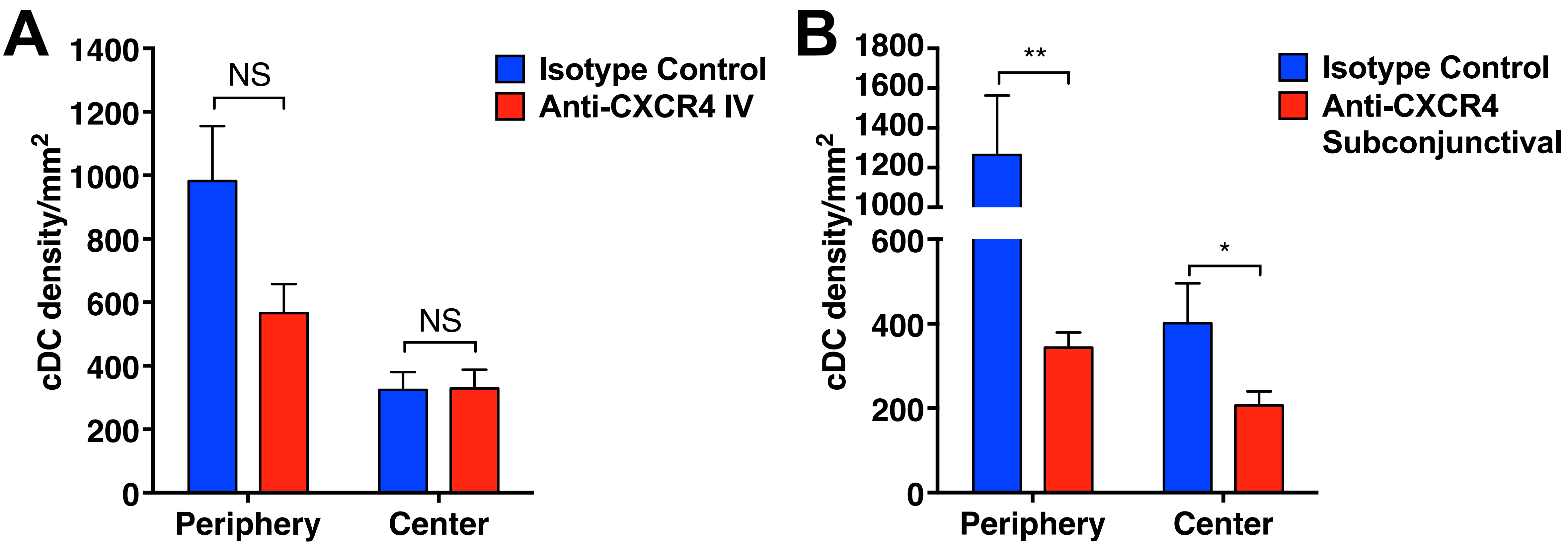
A
B
cDC density/mm2
1400
1200
1000
800
600
400
200
0
NS
NS
Periphery
Center
Isotype Control
Anti-CXCR4 IV
1800
1600
1400
1200
1000
600
400
200
0
**
*
Isotype Control
Anti-CXCR4
Subconjunctival

Supplemental Figure 2. Effect of local and systemic CXCR4 blockade on cDC infiltration into the inflamed cornea. Administration of anti-CXCR4 neutralizing antibody into inflamed mice via intravenous (i.v.) (A) or subconjunctival (B) injection 30 minutes prior to adoptive transfer of fluorescently labeled cDCs resulted in decrease in cDCs found in cornea 24 hours after, compared to isotype control. Data are shown as mean ± SEM, $*p<0.05$, $**p<0.01$, n=5-6 / group.
